# Racial Differences in Cumulative Disadvantage Among Women and Its Relation to Health: Development and Preliminary Validation of the Cumulative Stress Inventory of Women's Experiences

**DOI:** 10.1089/heq.2021.0038

**Published:** 2022-06-15

**Authors:** Kenzie Latham-Mintus, Tess D. Weathers, Silvia M. Bigatti, Amy Irby-Shasanmi, Brittney-Shea Herbert, Hiromi Tanaka, Lisa Robison, Anna Maria Storniolo

**Affiliations:** ^1^ Department of Sociology, IUPUI, Indianapolis, USA.; ^2^ Department of Social and Behavioral Sciences, IU Fairbanks School of Public Health at IUPUI, Indianapolis, USA.; ^3^ Medical and Molecular Genetics, Indiana University, Indianapolis, USA.; ^4^ Hematology/Oncology, Indiana University, Indianapolis, USA.

**Keywords:** stress, cumulative disadvantage, race, health

## Abstract

**Background:**

Cumulative disadvantage (CD) is a measure of accumulated social, economic, and person-related stressors due to unequal access to resources and opportunities, which increases a person's biological risk for disease. The purpose of this research was to develop an instrument tailored to women's experiences that had intervention and translational potential. In addition, we explored whether CD contributed to racial health disparities among black and white women.

**Methods:**

In-depth life course interviews were used to assess stressful experiences of 15 black and 15 white women. Using information from the interviews, we developed the Cumulative Stress Inventory of Women's Experiences (CSI-WE) as a quantitative instrument to measure stressful life experiences from childhood to adulthood. The CSI-WE was then administered to the original 30 women for validation and feedback.

**Results:**

Qualitative and quantitative assessments were highly correlated, which suggested that the CSI-WE reliably captured the experiences of the interviewed women. Black participants reported significantly higher numbers of childhood and adult stressors, more acute adulthood and lifetime stressors, and worse adult physical self-rated health.

**Conclusions:**

This study supports the preliminary validity of an instrument that once fully validated may be used in future studies to elucidate the experiences of CD among black and white women and examines how these experiences relate to perceived and objective health status.

## Introduction

Cumulative disadvantage (CD) refers to the *accumulating* influence of social, behavioral, and biological processes and their interplay on health over the life course.^1–4^ A tenet of CD is that early life advantage or disadvantage leads to differential exposures to health-detracting risk factors such as adverse life events.^2,3,5^ Race/ethnicity, socioeconomic status (SES), and gender shape individuals' exposure to social, behavioral, and biological risk even before birth.^6–8^ We conceptualize CD as a measure of *accumulated* social, economic, and person-related stressors due to unequal access to resources and opportunities, increasing a person's biological risk for disease.^4,9^ Social inequalities “get under the skin” via physiological damage from the sustained adrenal response to stress, causing increased susceptibility to disease and debilitation.^3,4,10,11^ In this way, stress is a driving force behind health disparities.^5,10,12,13^

Women in the United States, particularly women of color, experience unequal health across the life course.^14^ Structural racism and sexism contribute to constrained education and economic opportunities and reduced access to quality neighborhoods, housing, and health care.^14^ Discrimination increases exposure to social stressors and affects health, with black women experiencing “multiple jeopardy” and more severe health penalties than white women.^15–17^ In addition, cultural racism contributes to stigma and prejudice often invoking health-detracting psychological responses (e.g., stereotype threat and internalized racism).^18^ Discrimination represents a durable psychosocial stressor that shapes health outcomes including self-rated health.^18,19^

Kreiger's^6^ ecosocial theory helps understand the pathways leading to biological expressions of social inequality and health disparities. It emphasizes the need to examine disparities within a sociohistorical context; instruments designed to assess CD should reflect the lived experiences of women of color. Identifying which dimensions of disadvantage are most harmful, as well as who experiences them and under what conditions, may be the key to understanding health disparities.

This area of research has been hampered by the lack of instruments reflecting the unique social stressors experienced by women and women of color. It is crucial to expand beyond singular factors such as SES or trauma and measure the *multitude of life course events and their many dimensions*. To our knowledge, the Cumulative Stress Inventory of Women's Experiences (CSI-WE) is the first instrument designed to assess CD experiences among black and white women with input from participants. Other assessments of CD (see Albert et al.)^20^ miss important information because the social stressors included are based on researchers' assumptions about what events are significant. Allowing black and white women to tell their stories enabled us to capture stressors overlooked in the past.

Given the limitations of existing instruments, our purpose was to develop an instrument to explore racial differences in exposure to social stressors associated with CD among women to understand the complex biopsychosocial processes that contribute to racial health disparities. Our specific aims, focused on black and white women, were as follows: (1) use innovative, mixed-method techniques to develop and evaluate a measure of CD; and (2) examine CD experiences among black and white women and explore differences.

## Methods

### Study design

**FIG. 1.**
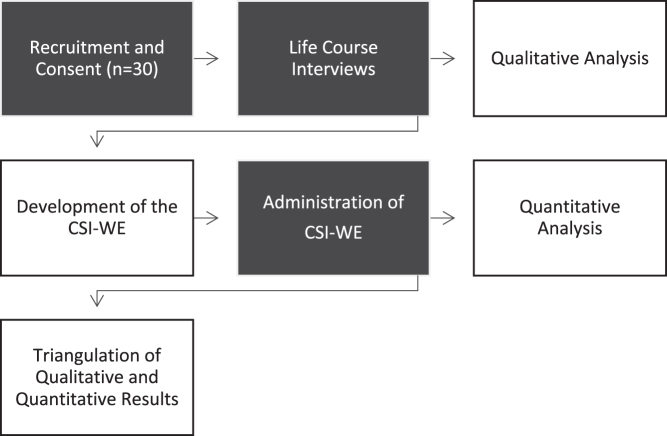
Study design.

We used an exploratory sequential mixed-methods design with a sample of 30 women. In-depth, life course interviews were conducted. Results were used to develop and test the quantitative CSI-WE.^21^ We analyzed results from the qualitative interviews and the CSI-WE together to achieve our aims. Figure 1 demonstrates each step in the design.

### Recruitment and consent

Following approval from the university Institutional Review Board, we recruited a purposeful sample of 15 white and 15 black women from among women who had donated normal breast tissue to the Komen Tissue Bank (KTB). The KTB e-mailed black and white donors willing to be contacted for research studies. The e-mail explained the study and instructed them to contact us if interested. Those who contacted us answered a series of questions to confirm eligibility.

The eligibility criteria were as follows: (1) black/African American or white; (2) female; (3) 25–50 years old; and (4) able to read, write, speak, and understand English. Black women who moved to the United States after the age of 5 were excluded to avoid life experiences not typical of the U.S. racial context; women who were postmenopausal or had a history of cancer or known genetic risk were excluded to avoid confounding of biomarker measures considered in parallel. Eligible persons provided verbal consent over the telephone and full consent before beginning the study. All 30 women completed the study procedures.

### Measurement

#### Qualitative life course interviews

All interviews were completed by two female interviewers who were race-concordant with interviewees. Participants were invited to share their “life story,” including main events, circumstances, or relationships during different periods of their life. These were semistructured in-depth interviews designed to investigate the participants' exposure to social conditions identified in extant literature as contributors to CD, and explore additional stressors perceived as impactful by participants.

The interview progressed through four life stages: childhood (birth through age 12), adolescent years (ages 13–18 or completion of secondary school), young adulthood (up to age 40), and current life (age 40 to current age). The interview guide questions, including the place/neighborhood where the participant lived, family life, school experiences and activities, impactful relationships (of any type), moves or relocations, motherhood, work experiences, other major events, significant sources of stress or challenge, and good things remembered about each phase of life. Interviews occurred in a private setting and lasted ∼1.5–2.5 h. Participants were paid $40. Interviews were audio-recorded for transcription and qualitative analysis.

#### Cumulative Stress Inventory of Women's Experiences

We developed the CSI-WE by drawing from existing instruments measuring aspects of objective and subjective CD, and improving and adding items based on the qualitative interviews. We first drew from national surveys or studies as well as previously validated instruments. National surveys/studies included the following: MIDUS II—the National Survey of Midlife Development in the United States,^22^ the Jackson Heart Study,^23^ and the National Survey of American Life.^24^ Validated instruments include the following: Adverse Childhood Experiences,^25^ traumas and stressful life events,^26^ chronic strains,^27^ discrimination^28^ (as combined by Turner and Avison),^12^ and additional measures of socioeconomic disadvantage, such as the Subjective Social Status Scale.^29^

After completing in-depth life course interviews and compiling existing CD instruments, we identified gaps and added or developed new items to capture stressful life experiences not addressed in existing instruments. Next, we added pertinent demographic and health information.

The final CSI-WE presents questions in the following topical sequence: (1) demographics, (2) current self-rated health and personal characteristics, (3) childhood demographics, (4) childhood stressors, (5) transition to adulthood, (6) romantic relationships, (7) relationships with family and friends, (8) parenthood (if applicable), (9) adulthood stressors, and (10) self-assessed impact of stress on health.

The Childhood Stressors section consists of a series of 57 questions “about stressful experiences (they) may have had as a child, up to age 18.” The Adulthood Stressors section consists of a series of 82 questions “about stressful experiences (they) may have had in (their) adult life, beginning at age 18 and continuing to the present.”

In line with the Jackson Heart Study's measure of acute and chronic stress,^30^ if respondents reported having had the experience, they were asked two follow-up questions. (1) How stressful was it at the time? [To measure acute impact.] (2) How stressful was it in the long run? [To measure chronic impact.] Response categories were as follows: not stressful (0), a little stressful (1), somewhat stressful (2), and very stressful (3). Participants were given the option to skip the question.

The CSI-WE was administered online to the 30 interview participants using REDCap (Research Electronic Data Capture), a secure web-based application designed to support data capture for research.^31^ Participants were paid $30 for completion of the online CSI-WE.

### Analysis

#### Analysis of qualitative interviews

Interviews were transcribed verbatim by research staff. To uncover potential processes and stressors not previously explored, while also recognizing processes and stressors already identified in the extant literature, we combined *a priori* and inductive methods.^32^ Two of the authors independently coded the interviews using Dedoose Version 8.0.35.^33^ First, the coders read all the transcripts. From the perspective of the participant, they excerpted all experiences described as stressful, disturbing, or painful. Second, qualitative categories were identified through systematic open coding. Codes were content analyzed to capture patterns that emerged. Next, smaller codes were combined into larger ones and coders identified connections between the codes to develop the CSI-WE.

Following completion of thematic coding, the two interviewers qualitatively rated the participants' overall stress load for two phases of life: childhood (childhood and adolescence combined) and adulthood. Both exposure and impact were considered during rating and were assigned using a 6-point scale as follows: (1) Very low, (2) Low, (3) Moderate, (4) High, (5) Very High, and (6) Extremely High. In assigning interviewer-rated CD, the interviewers considered the range of experiences observed among all participants. Following each interviewer's independent rating of the participants' stress load in these two large phases of life, they systematically reviewed the ratings together to reach consensus. To determine the ratings, the authors listed all the stressors that each woman experienced and when it occurred in her life. The gravity, duration, number, and frequency of stressors shaped the final rating score.

#### Analyses of specific aims

We examined how well the CSI-WE results were correlated with the interviewer-rated CD with bivariate correlations for childhood, adulthood, and lifetime measures. We also examined potential racial differences across each life stage. Given our small nonrandom sample, we used nonparametric tests to examine differences by race. Specifically, we conducted a series of Mann–Whitney *U*-tests, which is an alternative to the *t*-test, between black and white respondents to assess group differences. We examined the number of stressors, perceived acute stress (i.e., “How stressful was it at the time?”), perceived chronic stress (i.e., “How stressful was it in the long run?”), and perceived impact (i.e., “How much of an effect did stress have on your health?”) for childhood and adulthood, and we created a lifetime measure by summing each CD measure. These analyses were carried out in SAS 9.4.

## Results

Table 1 presents the sample characteristics by race. There were no statistically significant differences in age, education, marriage status, and parenthood status by race. In general, black women reported lower childhood and adult SES compared with white women.

**Table 1.** Comparison of Sample Characteristics by Race

	Total (***n***=30)	Black (***n***=15)	White (***n***=15)	***p***
Sociodemographic characteristics	Mean/percent^a^			
Age (years) (range=26–49)	38.7	38.7	38.7	0.983
Education (BA or higher=1)	80.0%	73.3%	86.7%	0.651
Married/partnered (yes=1)	63.3%	53.3%	73.3%	0.450
No children (yes=1)	40.0%	46.7%	33.3%	0.710
Adult SES				
Employment status				
Works full time (yes=1)	76.7%	80.0%	73.3%	1.000
Household income (range=1–11)	7.9	6.6	9.1	0.029
Owns home (yes=1)	66.7%	53.3%	80.0%	0.245
Subjective SES (10=top)	6.4	5.6	7.1	0.007
Childhood (SES)				
Mother's education (8=advanced degree)	5.5	4.4	6.6	0.007
Father's education (8=advanced degree)	5.8	5.0	6.6	0.022
Parents owned home (yes=1)	76.7%	53.3%	100.0%	0.006
No public assistance (yes=1)	70.0%	46.7%	93.3%	0.014
Perceived childhood SES (3=well off)	2.0	1.5	2.4	0.001
Very safe neighborhood (yes=1)	73.3%	60.0%	86.7%	0.215
No. of moves (5=6+ moves)	2.1	1.9	2.3	0.458
Health risk profiles				
Obese (yes=1)	40.0%	53.3%	26.7%	0.264
Current smoker (yes=1)	26.7%	13.3%	40.0%	0.215

^a^ Means listed for continuous variables, and percent listed for categorical variables; significance testing was completed using Fisher's exact test for categorical measures and Mann–Whitney *U*-test for continuous measures.

SES, socioeconomic status.

### Qualitative interview findings

The research team identified critical gaps in five domains: (1) social relationships/emotional support; (2) transition to adulthood; (3) identity formation; (4) unmet needs/lack of security; and (5) mental/emotional health. Forty-eight items were added to capture aspects of women's stressful life experiences not clearly addressed in existing instruments (Supplementary Table S1). Twenty items pertained to childhood life stage, while 28 items pertained to adulthood; 11 items were relevant in both life stages. Most of the items were newly developed by the team, while some were identified from existing instruments. Supplementary Table S2 shows an example item for each of the five domains, along with an exemplary quote.

### Comparison of qualitative and quantitative CD assessments

Table 2 compares the qualitative and quantitative CD assessments across childhood and adulthood. In general, a Pearson's *r* coefficient that exceeds 0.50 indicates a large effect size.^34^ Based on this, the interviewer-rated CD from the qualitative interviews was highly correlated with our measures in the CSI-WE. A combined measure of the qualitative interviewer rating (i.e., summing childhood and adulthood interview ratings with a range from 2 to 12) and an index of all individual stressors identified in the CSI-WE, representing the total cumulative burden over childhood and adulthood, were also strongly correlated (*r*(28)=0.62, *p*<0.001), although weaker compared with the childhood-only measures.

**Table 2.** Correlation Coefficients Comparing Qualitative Assessments of Cumulative Disadvantage with the Cumulative Stress Inventory of Women's Experiences

	No. of stressors	Acute stress	Chronic stress	Perceived impact
Childhood				
Interviewer rating of childhood CD	0.74^***^	0.74^***^	0.74^***^	0.46^*^
Adulthood				
Interviewer rating of adult CD	0.42^*^	0.41^*^	0.34^†^	0.21
Lifetime				
Summary of interviewer rating	0.62^***^	0.62^**^	0.62^***^	0.39^*^

† *p*<0.10; ^*^p<0.05; ^**^p<0.01; ^***^p<0.001.

CD, cumulative disadvantage.

A similar pattern emerged for subjective acute and chronic stress (i.e., weighting how stressful something was “at the time” and “in the long run” in the CSI-WE), where the childhood assessments were strongly correlated (i.e., exceeding 0.50), while adulthood assessments were moderately correlated (i.e., between 0.30 and 0.50). Overall, the interviewer-rated CD variable matched the experiences reported in the survey by the respondents—evidencing triangulation.

### Racial differences in CD and health

Table 3 explores differences in the distribution of CD and self-rated health among black and white women. Black women reported higher levels of CD, using multiple measures across the life course, despite having similar educational backgrounds and current work statuses. Specifically, as seen in Table 3, when compared with white participants, black participants had higher scores on acute adulthood stressors and acute lifetime stressors. They also reported higher median numbers of childhood and lifetime stressors, and lower adulthood physical health. All of these had effect sizes larger than 0.30, indicating at least a medium effect size. We did not see any significant differences in our measure of perceived stress impact—suggesting that perceived assessments may not reflect objective assessments of CD. We documented one significant difference in health; black women reported worse adult physical health than white women.

**Table 3.** Comparison of Cumulative Disadvantage and Self-Rated Health by Race

	Range	Total ***M*** (SD)	Black ***Mdn***	White ***Mdn***	Effect size ***r***	***p*** ^a^
Childhood CD						
Interviewer-rated childhood CD	1–6	3.0 (1.5)	3.00	2.00	0.20	0.269
**No. of childhood stressors^b^**	**1–37**	**10.9 (7.3)**	**12.00**	**7.00**	**0.37**	**0.041**
Acute childhood stress	3–125	36.5 (25.6)	39.00	29.00	0.26	0.158
Chronic childhood stress	2–86	31.4 (20.8)	36.00	22.00	0.28	0.124
Perceived health impact of childhood stress	0–3	1.7 (1.0)	2.00	2.00	0.10	0.574
Adulthood CD						
Interviewer-rated adulthood CD	1–6	3.5 (1.2)	4.00	3.00	0.17	0.352
Number of adulthood stressors	7–54	19.5 (9.9)	22.00	14.00	0.33	0.071
**Acute adulthood stress**	**28–168**	**69.7 (32.7)**	**84.00**	**48.00**	**0.38**	**0.038**
Chronic adulthood stress	11–101	46.4 (25.7)	60.00	28.00	0.34	0.062
Perceived health impact of adulthood stress	0–3	2.2 (0.7)	2.00	2.00	0.02	0.909
Lifetime CD						
Interviewer-rated lifetime CD	2–12	6.5 (2.5)	7.00	6.00	0.19	0.295
**No. of lifetime stressors**	**9–91**	**30.5 (16.2)**	**36.00**	**20.00**	**0.39**	**0.029**
**Acute lifetime stress**	**31–293**	**106.2 (54.4)**	**125.00**	**69.00**	**0.42**	**0.023**
Chronic lifetime stress	13–178	77.8 (42.9)	93.00	49.00	0.33	0.065
Perceived health impact of lifetime stress	1–6	3.9 (1.5)	4.00	4.00	0.09	0.610
Self-rated health						
Childhood mental health	1–5	3.3 (1.2)	3.00	3.00	0.00	0.983
Childhood physical health	2–5	4.1 (0.9)	4.00	4.00	0.01	0.946
Adulthood mental health	2–5	3.4 (0.8)	3.00	3.00	0.03	0.856
**Adulthood physical health**	**2–5**	**3.4 (0.8)**	**3.00**	**4.00**	**0.36**	**0.048**
Average lifetime mental health	1.5–5	3.3 (0.9)	3.50	3.00	0.04	0.229
Average lifetime physical health	2.5–5	3.8 (0.7)	3.50	4.00	0.22	0.833

^a^ Based on Mann–Whitney *U*-test.

^b^ Bolded variables indicate a *p*-value less than 0.05.

Table 4 displays the findings related to self-rated health and CD. Among the measures of self-rated health, there was a consistent inverse correlation between self-rated childhood mental health and our measures of CD. Interestingly, only chronic childhood stress was correlated with self-rated childhood physical health. In adulthood, chronic stress and perceived impact were both correlated with self-rated adult mental health. Number of stressors, acute stress, and chronic stress were all inversely correlated with self-rated adult physical health. All of the lifetime measures of CD were inversely correlated with average lifetime self-rated mental health, whereas only the subjective assessments of acute and chronic stress were inversely correlated with average lifetime self-rated physical health—perhaps underscoring the additional predictive capacity of subjective assessments.

**Table 4.** Correlation Coefficients for Self-Rated Health and Cumulative Disadvantage

	No. of stressors	Acute stress	Chronic stress	Perceived impact	Interviewer-rated CD
Childhood CD					
Self-rated mental health	−0.56^**^	−0.53^**^	−0.65^***^	−0.66^***^	−0.64^***^
Self-rated physical health	−0.24	−0.19	−0.42^*^	−0.27	−0.24
Adulthood CD					
Self-rated mental health	−0.27	−0.24	−0.30^†^	−0.50^**^	0.11
Self-rated physical health	−0.37^*^	−0.42^*^	−0.44^*^	−0.04	0.03
Lifetime CD					
Self-rated mental health	−0.50^**^	−0.47^**^	−0.55^**^	−0.67^***^	−0.45^*^
Self-rated physical health	−0.28	−0.32^†^	−0.42^*^	−0.23	−0.20

† *p*<0.10; ^*^p<0.05; ^**^p<0.01; ^***^p<0.001.

## Discussion

Our specific aims were to develop an instrument to assess CD using innovative mixed-methods techniques, evaluate the instrument, examine CD experiences among black and white women, and explore differences between groups and the relationships among race and self-reported health. Using in-depth life course interviews and the extant literature, we developed the CSI-WE to thoroughly capture multiple dimensions of CD among women. We identified critical gaps in the literature and added unique items to address these gaps. Specifically, we added items that reflected social relationships/support, identity formation, unmet needs/security, transition to adulthood, and mental/emotional health. These were based on the experiences of the women interviewed and reflect some of the unique stressors women experience in the United States.

These items provide a better understanding of the *cumulative* burden of stressful life events experienced by women, and how they differ by race. A large study (*n*=25,062) of cumulative psychosocial stressors in older women, using participants from the Women's Health Study, found that black women had the highest cumulative psychosocial stressors and white women the lowest.^35^ However, these factors tend to be studied at determined time points, not across the life course, and mostly specific to certain settings. For example, college-educated black women working in mostly white environments find that they must shift identity to succeed.^36^ Similarly, black female students in science, technology, engineering and mathematics (STEM) fields are aware of their intersectional identities and the associated risks and benefits. The CSI-WE will allow exploration of these experiences throughout the life course.^37^

We evaluated the CSI-WE by comparing the concordance between the qualitative and quantitative assessments of CD. There was strong evidence of triangulation. We were also able to explore in-depth the CD experiences of black and white women. In line with previous research related to the greater CD burden experienced by racial and ethnic minorities,^38^ black women experienced greater CD relative to white women. Although this finding is not new, our research underscores the disparate circumstances that lead to greater exposure to stressful life events among black women. We found statistically significant differences in the number of childhood and lifetime stressors and observed high burdens of acute adult and lifetime stress among black women. Black women also reported worse adulthood physical health than white women.

Although childhood has been described as a critical period for social and biological development, which is linked to the biological risk of disease,^39,40^ our research suggests that, among black women, these stressors spill into adulthood. Despite similar education backgrounds, the scale of difference between black and white women experiencing high CD was substantial.

These findings, which further support the need for the CSI-WE, are supported by extant literature. Among older adults, black women experience more ongoing stressors compared with white women, even after controlling for SES.^41^ In a comprehensive “crib to coffin” exploration of racism-related stress, Jones et al. delineate the many ways in which stressors challenge black women throughout their life course.^42^

There are several limitations worth noting. Our sample was highly educated and drawn from an urban area, which not only limits generalizability, but most likely underestimates racial differences. Although this is a major limitation, it highlights the need for more research. A more representative sample may show even larger disparities in CD. Our sample only included black and white women. Future research should refine this and other instruments to include the experiences of other women of color. Due to the small sample, we were unable to confidently report differences between groups in other areas where there was a medium effect size but not statistical significance. These included number of adulthood stressors, chronic adulthood stressors, and chronic lifetime stressors. This suggests that future research using our CSI-WE with larger samples may show even more differences between groups than reported here, and in this way suggests clear avenues for prevention and intervention efforts.

## Conclusion

We uniquely contribute to the literature by using a mixed-methods approach to develop an inventory to assess CD among black and white women. Our findings showed significant differences in lifetime experience of stress between groups in both childhood and adulthood, disadvantaging black women. This work has important implications including providing evidence of the vastly different CD experiences among black and white women and the preliminary development of an instrument that once fully validated may be used in future studies to guide intervention. To truly further our understanding, we need instruments that accurately reflect the lived experiences of racial and ethnic minorities. We will continue to revise the CSI-WE so that we are able to depict the range of stressors experienced by all women as an important step to understanding the racial and ethnic health disparities.

## Supplementary Material

Supplemental data

Supplemental data

## Abbreviations Used

CD: cumulative disadvantage
CSI-WE: Cumulative Stress Inventory of Women's Experiences
SES: socioeconomic status
KTB: Komen Tissue Bank
