# Supplementary material for: Racial Differences in Cumulative Disadvantage Among Women and Its Relation to Health: Development and Preliminary Validation of the Cumulative Stress Inventory of Women's Experiences
Source: Health Equity. 2022 Jun 15;6(1):427–34. doi: 10.1089/heq.2021.0038 (PMC9257543; doi:10.1089/heq.2021.0038)
Supplement: Supplemental data [file Suppl_TableS1.docx]

Supplemental Table 1. Description Interview-Informed CSI-WE Items

| **Domain** | **Item** | **Childhood** | **Adulthood** |
| --- | --- | --- | --- |
| Identity formation | Struggled to accept things about yourself… | X |  |
|  | Worry about being accepted… | X | X |
| Mental/  emotional health | Ever have too many sexual partners… |  | X |
|  | Ever have trouble remembering… | X |  |
|  | Ever regularly drink alcohol or use drugs… | X | X |
|  | Ever struggled with an addiction… |  | X |
|  | Ever struggled with infertility… |  | X |
|  | Family secret that really affected you… |  | X |
|  | Felt like too much is expected… |  | X |
|  | Have any lasting regrets… |  | X |
|  | Have any unmet important goals… |  | X |
|  | Kept a secret for an extended period… |  | X |
|  | Serious mental or emotional problems… | X | X |
|  | Seriously consider/attempt suicide… | X | X |
|  | Struggled to do everything you need to… |  | X |
|  | Weighed down by responsibilities… |  | X |
| Social relationship  /emotional support | Ever felt abandoned by parent… | X |  |
|  | Imbalanced relationships… |  | X |
|  | Lack love or support… | X | X |
|  | Live w/ someone easily angered… | X | X |
|  | Live w/ someone w/ an addiction, aside from drugs/ alcohol… | X | X |
|  | Ongoing care for a sick, limited, or frail… |  | X |
|  | Ongoing toxic relationship… | X | X |
|  | Spouse/partner was controlling... |  | X |
|  | Thought current relationship would end… |  | X |
|  | Worried about child/ren serious problems… |  | X |
|  | Worried about child/ren very unhappy… |  | X |
| Adulthood  Transition | Take on responsibilities… | X |  |
| Unmet needs/lack of security | Ever had to work multiple jobs or long hours… |  | X |
|  | Ever kicked out of your family home… | X |  |
|  | Family ever felt threatened… | X |  |
|  | Family ever struggle to pay bills… | X | X |
|  | Family gone without basic needs… | X | X |
|  | Feel unsafe or threatened at school… | X |  |
|  | Live in a home that was harmful… | X | X |
|  | Live in an overly crowded home or no bed… | X |  |
|  | Move around so much lacked stability… | X |  |
