# Supplementary material for: Racial Differences in Cumulative Disadvantage Among Women and Its Relation to Health: Development and Preliminary Validation of the Cumulative Stress Inventory of Women's Experiences
Source: Health Equity. 2022 Jun 15;6(1):427–34. doi: 10.1089/heq.2021.0038 (PMC9257543; doi:10.1089/heq.2021.0038)
Supplement: Supplemental data [file Suppl_TableS2.docx]

Supplemental Table 2. Example Items and Quotes for Each Domain

| Domain | Example Item | Exemplary Quote |
| --- | --- | --- |
| Identity formation | Were there things about yourself that you struggled to accept or thought others close to you did not accept? | *" ….. at this point my parents still didn't know any of this. ……. the priest at church preached on homosexuality destroys the family. ………. my relationship with my parents got really strained then, especially my mom. She would [say] …..I'm just worried about your salvation, and all this kind of stuff, and I'm just like eye roll. It's like on the one hand, I'm eye rolling and making jokes with my friends, but on the other hand I'm devastated."* |
| Mental/ emotional health | Have you ever kept a secret for an extended period of time from those close to you that was a weight or burden? | *" I was in kindergarten, ……. I don’t remember all the details I just remember being pulled into the closet .... I’m not sure exactly how many times it happened, but I never told anyone. I think for probably out of fear for him. I know that if I had told my father, he probably would have killed him. ... But I didn’t know until I started therapy that that has followed me my entire life, although I never really knew it, it was."* |
| Social relationships/ emotional support | Did you ever live with someone who got easily angered or was there so much fighting/arguing that you often felt like you were "walking on eggshells"? | *"You never knew what you were going to walk into that morning when she'd come and wake you up. So as a kid it was very confusing. As a teenager it was also, like, I felt like I was walking on eggshells all the time because you never knew what was going to upset her."* |
| Adulthood Transition | Did you take on responsibilities to care for yourself, sibling(s), or other family members because the person(s) who raised you did not do it? | *" I pretty much had to raise my brother. So my childhood after he was born, that's why I said, it was fine until he was born and then after he was born it was like I no longer had a childhood. I had to be a young adult: I had to cook, I had to clean, I had to take care of him. I got tired of doing it so I started running away from home. I started running away from home when I was 13."* |
| Unmet needs/ lack of security | Did you/your family move around so much that you lacked a sense of stability and security? | *"My childhood between birth and 12 years old, I call that my nomadic type of life back then....I don't remember anything of the house that I was born in……….I lived there, I lived with [various different aunts] ….., but the crazy part about that situation, that was just really, REALLY tough time for me. Particularly academically.... we slept on the floor, which I felt was so traumatic, I felt awful.”* |
